# An Experimental Platform for Real-Time Students Engagement Measurements from Video in STEM Classrooms

**DOI:** 10.3390/s23031614

**Published:** 2023-02-02

**Authors:** Islam Alkabbany, Asem M. Ali, Chris Foreman, Thomas Tretter, Nicholas Hindy, Aly Farag

**Affiliations:** 1 Electrical and Computer Engineering Department, University of Louisville, Louisville, KY 40292, USA; 2 College of Education and Human Development, University of Louisville, Louisville, KY 40292, USA; 3 Department of Psychology, College of Charleston, Charleston, SC 29424, USA

**Keywords:** student engagement, AI, behavioral engagement, emotional engagement

## Abstract

The ability to measure students’ engagement in an educational setting may facilitate timely intervention in both the learning and the teaching process in a variety of classroom settings. In this paper, a real-time automatic student engagement measure is proposed through investigating two of the main components of engagement: the behavioral engagement and the emotional engagement. A biometric sensor network (BSN) consisting of web cameras, a wall-mounted camera and a high-performance computing machine was designed to capture students’ head poses, eye gaze, body movements, and facial emotions. These low-level features are used to train an AI-based model to estimate the behavioral and emotional engagement in the class environment. A set of experiments was conducted to compare the proposed technology with the state-of-the-art frameworks. The proposed framework shows better accuracy in estimating both behavioral and emotional engagement. In addition, it offers superior flexibility to work in any educational environment. Further, this approach allows a quantitative comparison of teaching methods.

## 1. Introduction

**Figure 1:**
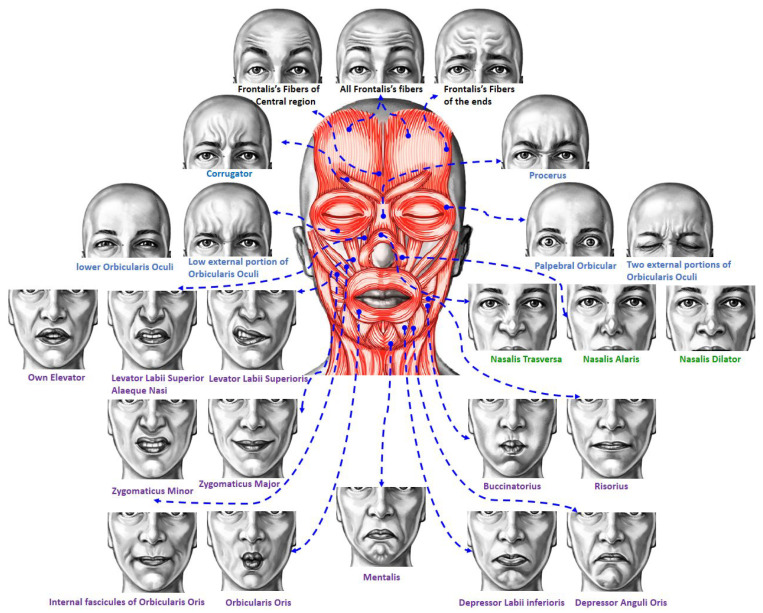
Human facial muscles that are responsible for different expressions. This illustration was designed using images generated by ARTNATOMIA [19].

The human face is an important tool for nonverbal social communication. Therefore, facial expression analysis is an active research topic for behavioral scientists. Due to its broad impact on several applications, such as pain assessment [1], diagnosis and treatment for autistic children [2] and detecting their emotional patterns [3], detecting distracted drivers [4], measuring students’ engagement [5], and human–computer interaction [6], in this work, we focus on student engagement. Facial expression analysis has attracted significant attention in the medical image processing community. There were early trials to study facial expressions. In 1862, G. Duchenne electrically stimulated facial muscles and concluded that movement of the muscles around the mouth, nose, and eyes constitute the facial expressions [7]. To express an internal emotional state, a person moves a set of facial muscles; see Figure 1.

Despite the urgent demand for graduates from science, technology, engineering, and mathematics (STEM) disciplines, large numbers of U.S. university students drop out of engineering majors [8]. Nearly one-half of students fail to complete an engineering program at the University of Louisville, which is consistent with national retention rates at large, public institutions [9,10]. This number is even higher for at-risk women, racial and ethnic minorities, and first-generation college students [11,12]. The greatest dropout from engineering occurs after the first year, following standard gateway mathematics courses such as calculus [13,14]. Dropout from the engineering major is strongly associated with performance in first-year mathematics courses [10,13]. Part of the difficulty, not limited to engineering, is the transition from secondary to college education in mathematics. Students often retain and apply only a surface-level knowledge of mathematics [15]. In addition, socio-psychological factors, such as perceptions of social belonging, motivation, and test anxiety, predict first-year retention [13,16,17,18].

The ability to measure students’ engagement in an educational setting may improve their retention and academic success. This ability may reveal disinterested students or which segments of a lesson cause difficulties. The main goal of the proposed work is to provide the instructors with a tool that could help them in estimating both the average class engagement level and the individuals’ engagement levels while they give lectures in real-time. This system could help the instructors to take actions to improve students’ engagement. Additionally, it could be used by the instructor to tailor the presentation of material in class, identify course material that engages and disengages with students, and identify students who are engaged or disengaged and at risk of failure.

Currently, feedback on student performance relies almost exclusively on graded assignments, with in-class behavioral observation by the instructor as a distant second. In-class observation of engagement by the instructor is problematic because he/she is primarily occupied with delivering the learning material. Indeed, adaptive learning environments allow free-form seating, and the instructor may not be able to have direct eye contact with the students. Even in traditional classroom seating, an instructor would not be able to observe a large number of students while lecturing. Therefore, it is practically impossible for the instructor to watch all students all the time while recording these observations student by student and correlating them with the associated material and delivery method. Moreover, these types of feedback are linked to the in-class environment. In an e-learning environment, the instructor may lose any feedback to sense student engagement. Performance on assignments can also be ambiguous. Some students can be deeply engaged yet struggling, whereas other students can be only minimally engaged; both groups end up with poor performance. Other students may manage good performance while lacking a deeper understanding of the material, e.g., merely studying to memorize an exam without engagement in the learning process.

**Figure 2:**
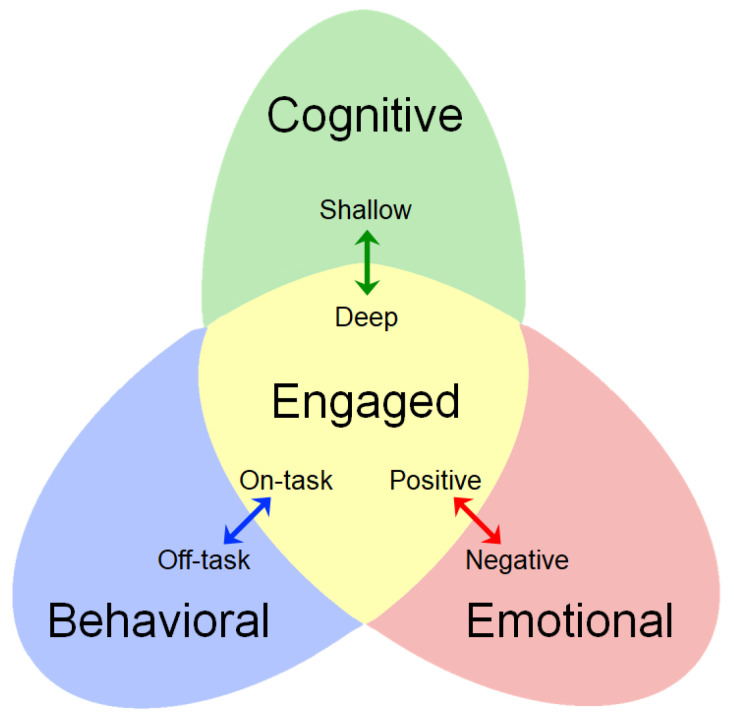
A conceptual framework linking on-task/off-task behavioral, positive/negative emotions, and deep/shallow cognitive engagement.

The education research community has developed various taxonomies describing student engagement. After analyzing many studies, Fredricks et al. [20] organized engagement into three categories. Behavioral engagement includes external behaviors that reflect internal attention and focus. It can be operationalized by body movement, hand gestures, and eye movement. Emotional engagement is broadly defined as how students feel about their learning, learning environment, teachers, and classmates. Operationalization of emotional engagement includes expressing interest, enjoyment, and excitement, all of which can be captured by facial expressions. Cognitive engagement is the extent to which the student is mentally processing the information, making connections with prior learning, and actively seeking to make sense of the key instructional ideas. The two former engagement categories can be easily sensed and measured. Cognitive engagement is generally less well-defined than the other two modes of engagement and is more difficult to externally operationalize due to its internalized nature [21,22]. The three components (shown in Figure 2) that comprise student engagement are behavior, emotion, and cognition [20]. These components work together to fully encompass the student engagement construct, and each component has been found to contribute to positive academic outcomes (e.g., [20,23]).

One of the significant obstacles to assessing the effect of engagement in student learning is the difficulty of obtaining a reliable measurement of engagement. Using biometric sensors (such as cameras, microphones, heart rate wristbands sensors, and EEG devices) is a more dynamic and objective approach for sensing. This work focuses primarily on measuring the emotional and behavioral components of the engagement and on designing a biometric sensor network and technologies for modeling and validating engagement in various class setups.

### Literature Review

The literature uses a variety of terms for instructional approaches (e.g., active vs. passive; hands-on vs. passive; minds-on vs. shallow receptive mental modes) that could potentially lead to variation in cognitive engagement. Table 1 provides a summary of the psychological constructs [24,25,26,27,28] for the three types of engagement, which could be used to devise a computational counterpart to automate engagement monitoring.

Despite the advances in machine recognition of human emotion, there have been a small number of studies of facial expressions related to learning-centered cognitive affective states. Computer vision methodology can unobtrusively estimate a student’s engagement from facial cues; e.g., [5,29,30,31,32,33,34,35,36]. Such studies apply one or more of the following paradigms. Observation and annotation of affective behaviors, investigation of facial action units involved in learning-centered effect, and application of automated methods to detect affective states.

Kapoor and Picard [29] used a camera equipped with IR LEDs to track pupils and to extract other facial features: head nodding, head shaking, eye blinking, eye and eyebrow shapes, and mouth activities. Additionally, a sensing chair is used to extract information about the postures. Moreover, they recorded the action that the subject was doing on the computer. Then, a mixture of Gaussian processes combined all the information and predicted the current affective state. In their study, eight children (8–11 years) were enrolled. Children were asked to solve puzzles on a computer. For 20 min, the screen activity, side view, and frontal view were recorded. From the collected videos, 136 clips were extracted (up to 8 s long). Teachers were asked to observe and record the affective state at eight samples per second. The affective states under consideration were high, medium, and low interest, boredom, and “taking a break”. The recognition rates of an interest vs. disinterest SVM classifier (for 65 interest samples and 71 disinterest 71 samples) were 69.84% (using upper face information) and 57.06% (using lower face information). They got a 86.55% recognition rate by combining all information, not only the facial features, using a mixture of Gaussian processes.

To detect the emotions that accompany deep-level learning, McDaniel et al. [30] investigated facial features. The affective states under consideration were boredom, confusion, delight, flow, frustration, and surprise. To perform their study, they asked 28 undergraduate students to interact with AutoTutor. First, participants completed a pretest. Then, videos of the participants’ faces were captured while interacting with the AutoTutor system for 32 min. Finally, they completed a posttest. After that, the affective states’ annotation was done by the learner, a peer, and two trained judges. The ground truth of the data was obtained from the trained judges, with an interjudge reliability Cohen’s kappa of 0.49. After that, the data were sampled to 212 emotion video clips (3–4 s) with affective states of boredom, confusion, delight, frustration, and neutral. Finally, two trained coders coded participants’ facial expressions using Ekman’s Facial Action Coding System. They computed correlations to determine the extent to which each of the AUs was diagnostic of the affective states of boredom, confusion, delight, frustration, and neutral. Their analyses indicated that specific AUs could classify confusion, delight, and frustration from neutral, but boredom was indistinguishable from neutral.

In order to study the learning-centered effect, Grafsgaard et al. [32] used an automated facial expression recognition tool to analyze videos of computer-mediated human tutoring. They collected a dataset of 67 undergraduate students who are learned from an introductory engineering course using JavaTutor software. Participants took six sessions of 45 min. Each session started with a pretest; then came the teaching session and post-session surveys; and finally, there was the posttest. During the teaching session, database logs, webcam facial video, skin conductance, and Kinect depth video were collected. Two trained coders coded participants’ facial expressions using a Ekman’s Facial Action Coding System to annotate the data. They recorded the five most frequently occurring AUs (1, 2, 4, 7, and 14). The authors used the CERT toolbox [37] to extract these 5 AUs automatically. Additionally, they computed the normalized learning gain from the posttest and the pretest scores. They claimed the following conclusions: outer brow raise (AU2) was negatively correlated with learning gain. Brow lowering (AU4) was positively correlated with frustration. Mouth dimpling (AU14) was positively correlated with both frustration and learning gain. Additionally, facial actions during the first five minutes were significantly predictive of frustration and learning at the end of the tutoring session.

Whitehill et al. [33] introduced an approach for automatic recognition of engagement from students’ facial expressions. They claimed that human observers reliably agree when discriminating low and high degrees of engagement (Cohen’s k = 0.96). This reliability decreased to k = 0.56 for four distinct levels of engagement. Additionally, they claimed that static expressions contain the bulk of the information used by observers, not the dynamic expressions. This claim means that engagement labels of 10-s video clips can be reliably predicted from the average labels of their constituent frames (Pearson r = 0.85). They collected a dataset of 34 undergraduate students who trained using cognitive skill training software. Each session started with an explaining video (3 min). Then there was a pretest (3 min), a training video (35 min), and finally, a posttest. The participant’s face was recorded during the training. To annotate the data, the video frames were coded by seven labelers using a scale to rate the engagement: 1: not engaged, 2: nominally engaged, 3: engaged in the task, 4: very engaged, and X: unclear frame. Then, 24,285 frames were selected such that the difference between any two labelers did not exceed one, and no labeler assigned X to the frame. The “ground truth” label of a frame was the integer average of all labels. Gabor features were extracted from the detect face to generate a 40 × 48 × 48 feature vector. Then, four binary SVM classifiers were used to detect a level out of the four levels of engagement. Finally, a multinomial logistic regressor was used to combine the output of the four binary classifiers. They claimed that automated engagement detectors perform with comparable accuracy to humans.

Li and Hung [35] reported enhancement of student engagement by the fusion of facial expressions and body features. Fusion of more disparate data can also enhance engagement measures, such as video facial expression with wristband heart rate data [38], and posture with electrodermal activity data fusion [39]. The use of context was explored by Dhamija and Boult [40] in the area of online trauma recovery, and they and others have found significant evidence [33,38,41,42] that facial-expression-based estimation of engagement is nearly universal. Additional work by Svati and Boult [36] explored the influences of mood awareness on engagement classification, where the mood is the prevailing state of emotion independent of the current task, e.g., classroom learning. Emotion affects the domain in which facial expressions and other biometrics are collected, and the understanding of how emotion affects engagement serves to fine-tune the use of these biometrics.

Ahuja et al. introduced a framework to sense a set of engagement-related features (EduSense) [34]. They extracted facial landmarks and use them to find facial features such as head pose and smile detection. They also performed body segmentation and body-key-point extraction. They used this to extract features such as detection of hand raise and sit vs. stand detection. Furthermore, they performed speech detection to find the ratio between instructor speech time and student speech time.

Table 2 is a listing of various features used in recent literature. Table 3 is a comparison among various frameworks and experiments to measure student engagement in term of learning context, sensors, affictive state, dataset, and annotation. Our team developed a framework for measuring student engagement level using facial information for an e-leraning environment [5,43,44,45,46]. The main goal of this paper is to propose a framework to measure the student engagement level in the in-class environment. Estimating engagement level in the in-class environment is more complicated. Rather than the presence of only one target of interest (laptop screen) in the case of e-learning, there are multiple targets of interest in the in-class environment. The student may look at the instructor, the whiteboard, the projector, his/her laptop screen, or even one of his/her peers. Therefore, the framework should track where each student’s gaze is. Then, it should relate them together to estimate the students’ behavioral engagement.

## 2. Proposed Behavioral Engagement Module

Behavioral engagement consists of the actions that students take to gain access to the curriculum. These actions include self-directive behaviors outside of class, such as doing homework and studying; and other activities are related, such as shifting in the seat, hand movements, body movements, or other subs/conscious movements while observing lectures. Finally, one can participate cooperatively in in-class activities [72,73].

Head pose and eye gaze are the main metrics with which to measure the student’s behavioral engagement. By estimating the student’s point of gaze, it can be estimated whether he/she is engaged with the lecture. If the student is looking at his/her laptop or lecture notes, the whiteboard, the projector screen, or the lecturer, he/she is probably highly behaviorally engaged. If a student looks at other things, he/she is probably not engaged. In the proposed system, distracted and uninterested students are identified by a low behavioral engagement level regardless of the reason for this distraction. For a regular class setting with an assumption that students are in good health, this distraction is related to class content. On the other hand, a student’s illness can be detected by measuring the student’s vital signs using a wristband. Additionally, a student’s fatigue can be identified using his or her emotions. Moreover, other abnormalities, such as eye problems and nick movement problem, can be identified by the instructor at the beginning of the class. All these types of disengagement should not be included in class content evaluation.

**Figure 3:**
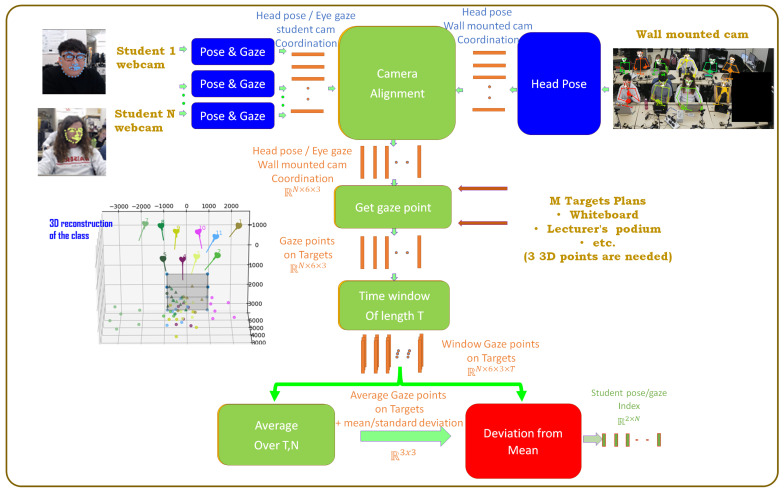
The proposed behavioral engagement framework.

In the proposed framework, two sources of video streams were used. The first source was a wall-mount camera that captured the whole class, and the second source was the dedicated webcam in front of each student. The proposed pipeline is shown in Figure 3. The first step in the framework is tracking key facial points and using them to extract the head pose [74]. It takes advantage of using a convolutional experts-constrained local model (CE-CLM), which uses a 3D representation of facial landmarks and projects them on the image using orthographic camera projection. This allows the framework to estimate the head pose accurately once the landmarks are detected. The resulting head pose could be represented in six degrees of freedom (DOF) (three degrees of freedom of head rotation (R)—yaw, pitch, and roll—and 3 degrees of translation (T)—X, Y, and Z). Eye gaze tracking is the process of measuring either the point of gaze or the motion of an eye relative to the head. The eye gaze could be represented as the vector from the 3D eyeball’s center to the pupil. In order to estimate the eye gaze using this approach, the eyelids, iris, and pupil are detected using the method in [75]. The detected pupil and eye location are used to compute the eye gaze vector for each eye. A vector from the camera origin to the center of the pupil in the image plane is drawn, and its intersection with the eye-ball sphere is calculated to get the 3D pupil location in world coordinates.

The wall-mounted camera provides the head pose only, as the faces size is too small to get accurate eye gaze from it, and the students’ cameras provide us with both head poses and eye gazes. Each camera provides the output in its world coordinates. Therefore, the second step is to align all the camera’s coordinates to get all students’ head poses and eye gazes in a common world-coordinate system. Given a well known class setup, the target planes could be found through one-time pre-calibration for the class. The intersections of the students’ head pose/eye gaze rays and the target planes are calculated. To eliminate noise, the feature was combined within a window of time of size T. Then, the mean point of gaze could be found on each plane in addition to the standard deviation for each window of time. The plane of interest in each window of time is the one with the least standard deviation of the students’ gaze. For each student, the student’s pose/gaze index could be calculated as the deviation of student’s gaze points from the mean gaze point in each window if time. This index is used to classify the average behavioral engagement within a window of time.

## 3. Proposed Emotional Engagement Detection Module

**Figure 4:**
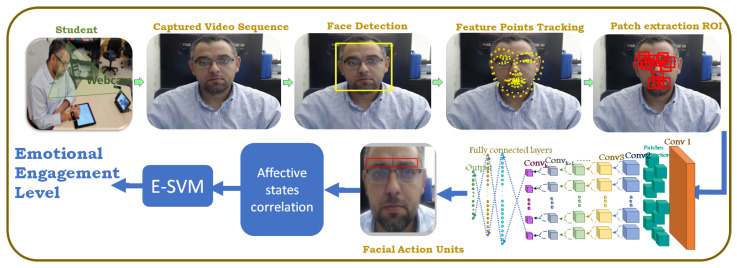
The proposed emotional engagement framework.

Emotional engagement is broadly defined as how students feel about their learning [76], learning environment [77], and instructors and classmates. Emotions include happiness or excitement about learning, boredom or disinterest in the material, and frustration due a struggle to understand [78]. In this section, a novel framework for the automatic measurement of the emotional engagement level of students in an in-class environment is proposed. The proposed framework captures the video of the user using a regular webcam; it tracks their faces throughout the video’s frames. Different features are extracted from the user’s face—e.g., facial landmark points and facial action units—as shown in Figure 4.

**Figure 5:**
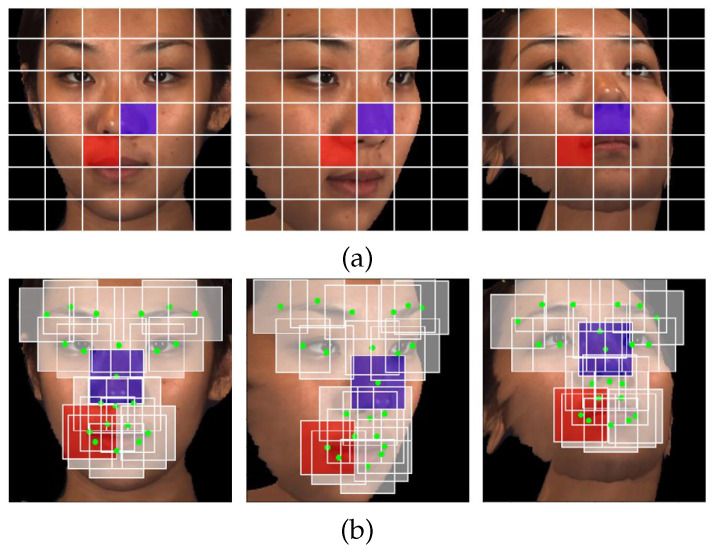
In the presence of pose, the uniform grid (**a**) suffers from a lack of correspondences (red and blue rectangles) due to displacement and occlusion. To minimize this lack of correspondence, facial landmarks are used to define a sparse set of patches (**b**).

It is logical to assume that a low measure of attentiveness indicated by the behavioral engagement component will not be enhanced by the emotional engagement classifier. Therefore, the application of the emotional engagement classifier is predicated on evidence of behavioral engagement in overall engagement estimation. To measure emotional engagement, the proposed module uses the extracted faces from previous steps to extract 68 facial feature points using our approach presented in [79]. This approach’s performance depends on a well-trained model. The current model was trained on the multiview faces 300 Faces In-the-Wild database [80], which has faces with multi-PIE (pose, illumination, and expression); therefore, the model performs well on different poses. Such a model allows the framework to estimate students’ engagement even if their faces are not front-facing. This helps student to sit freely on their seats without restrictions. Next, it uses our proposed method for action-unit detection under pose variation [81]. It uses the detected facial points to extract the most significant 22 patches to be used for the action-unit detection, as discussed in our work in [81]. This AU detection technique exploits both the sparse nature of the dominant AUs regions and semantic relationships among AUs. To handle pose variations, this algorithm defines patches around facial landmarks instead of using a uniform grid, which suffers from displacement and occlusion problems; see Figure 5. Then, it used a deep region-based neural network architecture in a multi-label setting to learn both the required features and the semantic relationships of AUs. Moreover, the weighted loss function is used to overcome the imbalance problem in multi-label learning. Then, the extracted facial action units are used to estimate the affective states (boredom, confusion, delight, frustration, and neutral) by correlations using McDaniel et al.’s method [30]. This method determines the extent to which each of the facial features is used to feed a support vector machine (SVM) to classify the students’ emotional engagement into two categories, emotionally engaged or emotionally non-engaged.

## 4. Experiments

### 4.1. Hardware Setup

**Figure 6:**
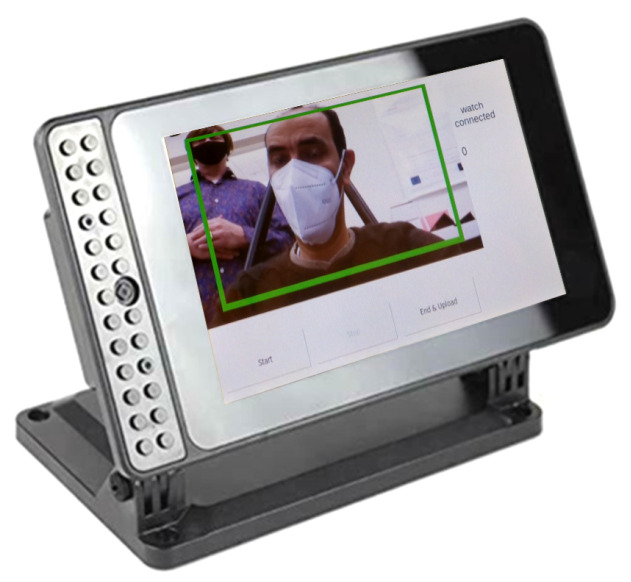
The student hardware module.

Using student webcams and machines to run the proposed client module raises many issues, especially with the huge variety that students have in terms of hardware and software. The camera quality cannot be guaranteed, and multiple versions of the software are needed to ensure that it runs on each operating system. Additionally, the student may fold his/her laptop and use it to take notes, which leads to the impossibility of capturing the student’s face. Therefore, a special hardware unit was designed and installed in the classroom to be used as our client module to capture students’ faces. This module is composed of a Raspberry Pi microcontroller connected to a webcam and a touch display; see Figure 6. The Raspberry Pi micro-controller runs a program that connects to the server, captures the video stream, applies the introduced pipelines to extract the feature vector, and sends that vector to the server. The program allows the students to adjust the webcam to ensure that the video has a good perspective of the face. This module is also used in the data collection phase. It captures a video stream of the student’s face during the lecture and processes it in real-time to obtain the required metric and send the features to a server/high-performance computing machine. The Raspberry Pi uses a TLS-encrypted connection to ensure students’ data security and privacy.

**Figure 7:**
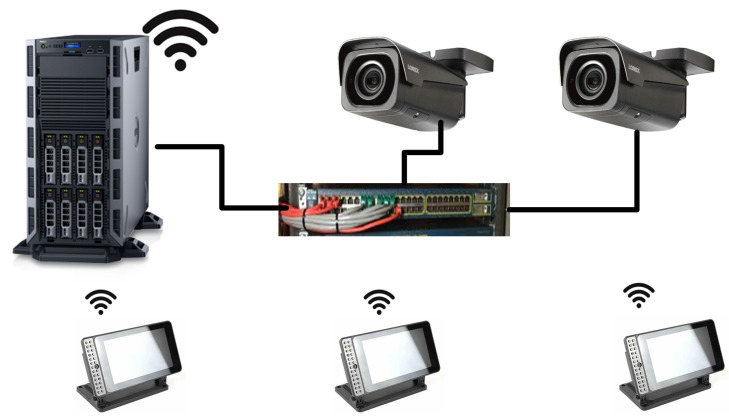
The proposed biometric sensor network.

**Figure 8:**
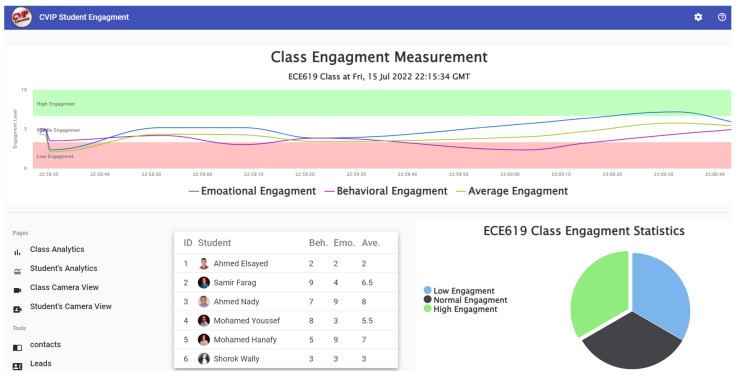
Instructor dashboard summarizes class students’ engagement in a clear and simplified way.

Our server is a high-performance computing machine that collects all the streams/features and classifies the engagement level in real time. The setup also includes 4K wall-mounted cameras to capture a stream of students’ faces to get their head poses. Additionally, the configuration provides high-bandwidth network equipment for both wire and wireless connections, see Figure 7. The server also provides the instructor with a web-based dashboard which allows the instructor to monitor the average class engagement level or the individual’s levels; see Figure 8. The instructor can monitor the dashboard on a separate screen without obstructing the dynamics of the class. The dashboard gives the instructor the average of class engagement in real-time. Thus, regardless of the class size, the dashboard is compact and has a simple illustration. In addition, more individual analysis can be shown offline after the class, if needed.

### 4.2. Data Collection

**Figure 9:**
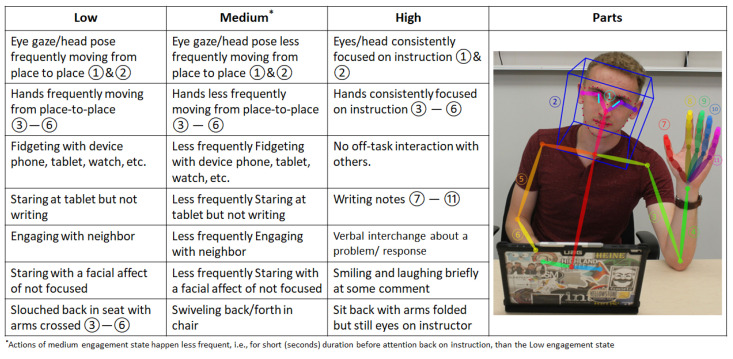
Dictionary of Tokens.

**Figure 10:**
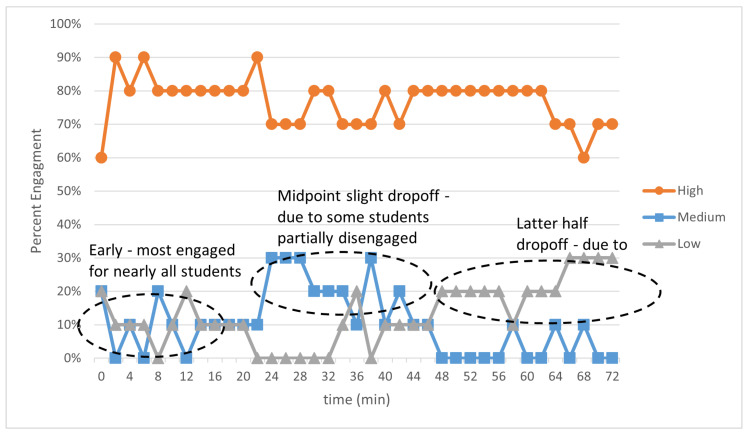
Engagement level of 10 students during a lecture.

The hardware described in the previous section was used to capture subjects’ facial videos while attending four lectures. The facial videos were recorded during the lectures. The collected dataset consists of 10 students of 300-level stem classes. These data were annotated by professorial educators. Each lecture is 75 min in length and was divided into 2-min windows, which resulted in 1360 samples. These data were annotated by education experts. Three engagement levels were defined using a set of tokens, which are summarized in Figure 9. A sample of the annotation during a lecture is shown in Figure 10. At the beginning of the lecture, most students were engaged. In the middle of the lecture, students’ engagement dropped somewhat due to some students partially disengaging. Later, at the end of the lecture, half dropped off, due to some students mostly disengaging. These results provide strong evidence for common observable behaviors and/or emotions that reflect student engagement.

### 4.3. Evaluation

**Figure 11:**
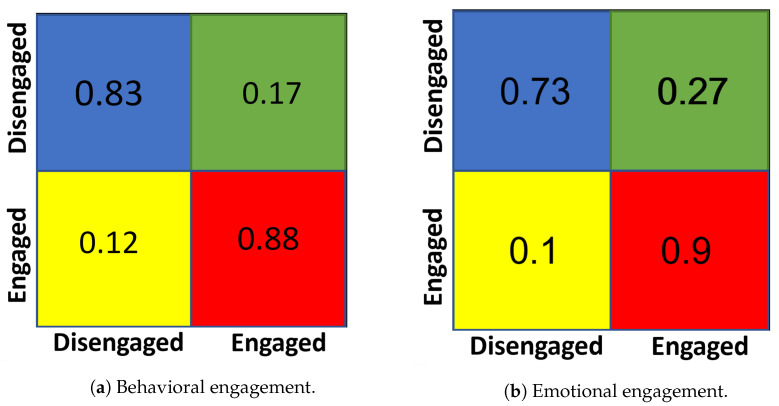
The confusion matrix of the proposed engagement classification.

A high-performance computing machine could run the proposed framework at a high frame rate of 10–15 fps, depending on the number of students in class. Raspberry Pi micro-controllers are able to run the proposed framework and process video stream to extract the individual students’ features (Head pose, Eye gaze, Action units) with a rate of 2–3 frames per second. Within a 2 min time window, we can get 240 processed feature vectors. The collected dataset was used to train support vector machine (SVM) classifiers to classify the engagement components (the behavioral and emotional engagement). The leave-one-out cross-validation technique was used for evaluation. The result-agreement ratios for the disengaged and engaged in terms of behavioral engagement were 83% and 88%, respectively. The agreement ratios for the disengaged and engaged in terms of emotional engagement were 73% and 90%, respectively. Figure 11 shows the confusion matrices of the proposed behavioral and emotional engagement classification.

## 5. Conclusions and Future Work

In this paper, a novel framework for automatically measuring the student’s behavioral and emotional engagement levels in the class environment was proposed. This framework provides instructors with real-time estimation for both the average class engagement level and the engagement level of each individual, which will help the instructor make decisions and plans for the lectures, especially in large-scale classes or in settings in which the instructor cannot have direct eye contact with the students.

More features should be captured to enhance the behavioral and emotional engagement modules. The streams from the 4K cameras will be used to capture students’ bodies, then extract their body poses and body actions. These actions will help the behavioral module to classify the students’ behavioral engagement. Additionally, to enhance emotional engagement, we consider adding some features such as heart rate variability (HRV) and galvanic skin response (GSK).

A large-scale dataset should be collected for more students who attend multiple courses during the entire semester. This will help in the process of training and evaluating both behavioral and emotional engagement measurement modules. It will also allow the emotional engagement measurement module to become more complicated by classifying chunks of video (time window) rather than individual frames.

Additionally, this work did not discuss the estimation of the third component of engagement, which is the cognitive engagement. Measuring this component is too complicated, and using a sensor such as an electroencephalogram (EEG) headset is very intrusive. A study to relate the measured behavioral and emotional engagement levels to the third component will be performed.

We tested the system in multiple classes, for which the lecture duration was 75 min. The system keeps track of students’ engagement over time. Thus, the time index helps an instructor with analyzing the engagement to evaluate the course content, while ignoring the beginning and end of the lecture. As future work, we plan to test the system in different classes with different durations and different settings. Moreover in this framework, data were collected in STEM classes for junior students at U.S universities. However, this system has a modular architecture; the more modules you add the better results you get. Thus, once the concept is proved and the technology is adopted, these modules can be minimized, and a smaller number of sensors can be used. Then, the technology can be delivered at a low cost, which makes it available in other places.

## Figures and Tables

**Table 1:** Psychological Constructs for the Three Types of Engagement.

TYPE OF ENGAGMENT	COGNITIVE (C)	BEHAVIORAL (B)	EMOTIONAL (E)
Psychological Construct	Levels of processing [24,25]	Targets of attention [26,28]	Affective context [27]
Engaged State	Deep processing	On-task attention	Positive affect
Disengaged State	Shallow processing	Off-task attention	Negative affect
External Operationalization	Not directly observable	Eye gaze, head pose, etc	Facial Action Coding System

**Table 2:** Features of interest along with motivating literature.

Feature	Engagement Component	Motivation
Body gestures and postures	Behavioral and Emotion	There are common observable behaviors and/or emotions, which reflect student engagement [47,48]. Student engagement can be estimated by the fusion of facial expressions and body features [35,49]. Fusion of facial expressions with wristband heart rate data and/or posture with electrodermal activity data shows dramatic improvement in student engagement [38,39]. Body movement, posture, and gesture indicate the affective states [50,51,52,53,54]. Gestures are associated with learning by indexing moments of cognitive instability and reflecting thoughts not yet found in speech [55,56].
Hand movement	Behavioral and Emotion	Hand-over-face gestures is a subset of emotional body language [57]. Frequency and quantity of hands raises is a good indicator of student participation [58]. Students who spontaneously gesture as they work through new ideas tend to remember them longer than those who do not move their hands [59].
Head movement and eye gaze	Behavioral	Head orientation has been shown to be a proxy for gaze attention [60,61,62,63], e.g., toward the instructor, educational materials, and other classroom foci. Attention is a pre-requisite for learning [64].
Facial Action Units (FACS)	Emotion	Facial information reveals students’ affective state, such as engagement [33] and frustration [32,65,66], and off-task behaviors [67]. Student engagement can be estimated by the fusion of facial expressions and body features [35,49].
Mood	Emotion	Mood awareness influences engagement classification [36].
Heart rate	Emotion	There are significant evidence [33,38,41,42] that facial expression estimation of engagement is nearly universal.
Graded homework and weekly exams	Cognitive	Cognitive engagement is challenging to directly monitor [68], especially during class sessions.

**Table 3:** Comparison of motivating literature frameworks settings.

Research Group	Learning Context	Information Source	Affective States	Annotators	Dataset
MIT Media Lab, 2005 [29]	A person Solving puzzles on PC	Camera (head-nod and head-shake, Eye blinks, Mouth activities); Chair (Posture features), OS (screen activity)	high, medium and low interest, bored, and “taking a break”	teachers	8 children
U. of Memphis 2007 [30]	An individual using AutoTutor system on PC	Camera (Manual Facial Action Coding) AUs 1, 2, 4, 7, & 14.	boredom, confusion, frustration, delight, and neutral	Self, peer, and 2 judges	28 undergraduate students
U. Massachusetts Amherst, Arizona State Emotion Sensors, 2009 [54]	An Individual using a multimedia adaptive tutoring system for geometry on PC	Camera (facial expression), Chair (Posture features), and mouse (increasing amounts of pressure), Wrist (skin conductance)	Confident, Frustrated, Excited, and Interested	Pretest, posttest, and a survey	38 HS and 29 female Undergrad students
NC State - 2013 [32]	An individual using JavaTutor on PC	Kinect (5 most frequently occurring AUS 1, 2, 4, 7, 14.)	Frustration and Learning game	Two FACS coders	67 undergraduate students
UCSD, MP Lab and Dept. of Psychology vsu, 2014 [23]	An Individual using a cognitive skills training software	Camera (Facial image)	Not engaged, Nominally engaged, Engaged, and Very engaged	7 Labelers	34 undergraduate students
Artificial Intelligence Department, UNED, Spain, 2014 [69]	An Individual solving Math. problems On PC	Camera (facial image); Kinect (video and processed information); OS (participant’s activities) Sensors (physiological signals) Solutions, questionnaires/observer report.	Excited/Unexcited, Concentrated/Distracted, Worried/Unworried, interested/Uninterested	Self-report, Psychoeducational expert	75 undergraduate
Learning Research Center, Pittsburgh Psychology and Education, 2015 [70]	A Finland high school 9th to 11th grade in regular classroom	Student- and teacher-report survey; students’ perceived value, importance, and level of enjoyment with school; scale students’ levels of stress, etc.	Emotional engagement, School burnout, and Depression symptoms	GPA/teachers report	362 students
Notre Dame, Florida State and Columbia, 2016 [67]	An Individual solving Physics problems On PC	Camera (nineteen AUS, head pose, and gross body movement)	Off-task, on-task, delight, boredom, concentration, confusion, frustration.	Baker-Rodrigo, Observation, Method	137 8th and 9th grade students
CVIP lab, University of Louisville (EITL) [5]	E-learning for undergraduate student	Student webCams (33 AU, Head pose, eye gaze)	No Face, Not engaged, Look engaged, Engaged, and Very engaged	Researchers	13 students
Carnegie Mellon University, (Edusense), 2019 [34]	Undergrads regular classroom setting	12 tripod mounted cameras, Front (Student), Back (Teacher)	Raw Features: Body pose, head pose, smile, mouth open, hand rise, sit vs. stand, student vs. teacher speech (time ratio)	-	25 students for training and 687 for evaluation
Carnegie Mellon University, (Classroom digital Twins), 2021 [71]	Undergrads regular classroom setting	2 Wall mounted cameras: Front (Student), Back (Teacher)	Raw Features: Head Pose, Gaze point	Controlled experiment, (marker)	8 participants
CVIP lab, University of Louisville (Our proposed)	Stem classes for undergrads, regular classroom setting	Wall mount Camera(Head pose), Student Processing unit (Head pose, eye gaze and Facial AUs)	Behavioral and Emotional Engagement	Education experts	10 students

## Data Availability

Data is unavailable due to students privacy.
